# Chest CT abnormalities in COVID-19: a systematic review

**DOI:** 10.7150/ijms.50568

**Published:** 2021-08-01

**Authors:** Ramy Abou Ghayda, Keum Hwa Lee, Jae Seok Kim, Seul Lee, Sung Hwi Hong, Kyeong Seok Kim, Kyeong Eon Kim, Jinhyn Seok, Hajeong Kim, Jangsuk Seo, Seungmin Lee, Ai Koyanagi, Louis Jacob, Lee Smith, Han Li, Andreas Kronbichler, Jae Il Shin

**Affiliations:** 1 Division of Urology, Brigham and Women's Hospital and Harvard Medical School Boston, MA 02115, USA; 2 Department of Global Health and Population, Harvard T.H. Chan School of Public Health, Boston, MA 02115, USA; 3 Department of Pediatrics, Yonsei University College of Medicine, Yonsei-ro 50, Seodaemun-gu, C.P.O Box 8044, Seoul 03722, Republic of Korea; 4 Department of Nephrology, Yonsei University Wonju College of Medicine, Ilsan-ro 20, Wonju 26426, Republic of Korea; 5 Yonsei University College of Medicine, Seoul, Republic of Korea; 6 Research and Development Unit, Parc Sanitari Sant Joan de Déu, CIBERSAM, Dr. Antoni Pujadas, 42, Sant Boi de Llobregat, 08830, Barcelona, Spain.; 7 ICREA, Pg, Lluis Companys 23, Barcelona, Spain; 8 Faculty of Medicine, University of Versailles Saint-Quentin-en-Yvelines, Montigny-le-Bretonneux, 78180, Versailles, France.; 9 The Cambridge Centre for Sport and Exercise Sciences, Anglia Ruskin University, Cambridge CB1 1PT, UK; 10 University of Florida College of Medicine, Gainesville, FL 32610, USA; 11 Department of Internal Medicine IV, Nephrology and Hypertension, Medical University Innsbruck, Innsbruck, Austria

**Keywords:** Coronavirus disease 2019 (COVID-19), Computed tomography (CT), Systematic review

## Abstract

Computed tomography (CT) of the chest is one of the main diagnositic tools for coronavirus disease 2019 (COVID-19) infection. To document the chest CT findings in patients with confirmed COVID-19 and their association with the clinical severity, we searched related literatures through PubMed, MEDLINE, Embase, Web of Science (inception to May 4, 2020) and reviewed reference lists of previous systematic reviews. A total of 31 case reports (3768 patients) on CT findings of COVID-19 were included. The most common comorbid conditions were hypertension (18.4%) and diabetes mellitus (8.3%). The most common symptom was fever (78.7%), followed by cough (60.2%). It took an average of 5.6 days from symptom onset to admission. The most common chest CT finding was vascular enlargement (84.8%), followed by ground-glass opacity (GGO) (60.1%), air-bronchogram (47.8%), and consolidation (41.4%). Most lung lesions were located in the lung periphery (72.2%) and involved bilateral lung (76%). Most patients showed normal range of laboratory findings such as white blood cell count (96.4%) and lymphocyte (87.2%). Compared to previous published meta-analyses, our study is the first to summarize the different radiologic characteristics of chest CT in a total of 3768 COVID-19 patients by compiling case series studies. A comprehensive diagnostic approach should be adopted for patients with known COVID-19, suspected cases, and for exposed individuals.

## Introduction

A cluster of patients presenting with pneumonia of unknown etiology was one of the first signs to trigger suspicion of a common epidemical etiology of cases, weeks before the identification of coronavirus disease 2019 (COVID-19), a novel coronavirus, as the culprit pathogen by the Chinese as well as the WHO authorities 1-3. Furthermore, healthcare workers have recognized that the virus was highly contagious to spread rapidly from person to person, in both symptomatic as well as asymptomatic individuals 4-6. Currently, the number of confirmed cases has been greatly dynamic, increasing daily across the globe with multiple confirmed deaths. Moreover, recently, new variants including delta are globally spreading, and the crisis is rising again despite vaccination efforts 7.

COVID-19 is caused by single-stranded RNA virus belonging to the family Coronaviridae in the order of Nidoviarles and is enveloped with a positive sense 8. Its respiratory manifestations are caused by the beta genre of this zoonotic virus, similar to the severe acute respiratory syndrome coronavirus (SARS-CoV) and Middle East respiratory syndrome coronavirus (MERS-CoV) 8. Transmission is achieved through large droplets generated by coughing or sneezing, as well as transport of the virus from surfaces into the mucosa of the mouth, nose, or eyes. Vertical transmission has also been reported 9. Clinically, signs and symptoms of the disease are variable and fall on a spectrum ranging from asymptomatic to multi-organ failure or unfortunate death 9. Respiratory manifestations are similar to other respiratory infections. They include but are not limited to cough, sore throat, and shortness of breath. In a subset of infected individuals, pneumonia and respiratory failure develop as part of severe complications, such as acute respiratory distress syndrome (ARDS) and acute lung injury, leading to most of the morbidities and mortalities 9. This latter severe manifestation of infection is thought to be due to the robust increase in inflammatory cytokines, including interleukins 2, 7 and TNF- α, among others 2, 9. One of the main diagnostic tools for lung involvement in the pathophysiology of the infection include computed tomography (CT) of the chest. In fact, given the high clinical sensitivity and specificity of chest CT, this imaging modality has been used in suspected infections with negative molecular testing 9, 10.

Until now, there have been several systematic reviews and meta-analyses on chest CT findings associated with COVID-19 11-15, but there are no comprehensive studies collecting all case series studies regarding the findings on chest CT in patients with confirmed COVID-19 and their association with clinical severity of viral infection. In this article, we show new perspectives on the most sensitive and specific CT imaging manifestations, the severity of the disease and the patient's comorbidities. Previous studies have shown variable radiologic signs and findings of unknown clinical significance and prognosis. Findings from this review can serve as a guide and add to the growing knowledge of clinical perspectives of this pandemic.

## Methods

### Literature search strategy and selection criteria

The Preferred Reporting Items for Systematic Reviews and Meta-analyses (PRISMA) guidelines were followed for this systematic review and meta-analysis. We searched PubMed, MEDLINE, Embase, Web of Science, and limited the search to human findings and included reports published in any language. The search terms used were as follows: “coronavirus 19”, “COVID-19”, “and 2019-nCoV”, “novel coronavirus 2019”, “CT”, and “computed tomography”.

We reviewed papers describing the findings on chest CT findings of COVID-19 positive patients during the extraction process. Because of the scarcity of evidence, papers without patient numbers were included. After the search process, we selected case series on the association of chest CT findings with the severity of the disease and patient mortality rates. In this process, we excluded *in vitro* or *in vivo* studies, genetic studies, and conference abstracts. The search was restricted to studies in humans.

**Figure 1:**
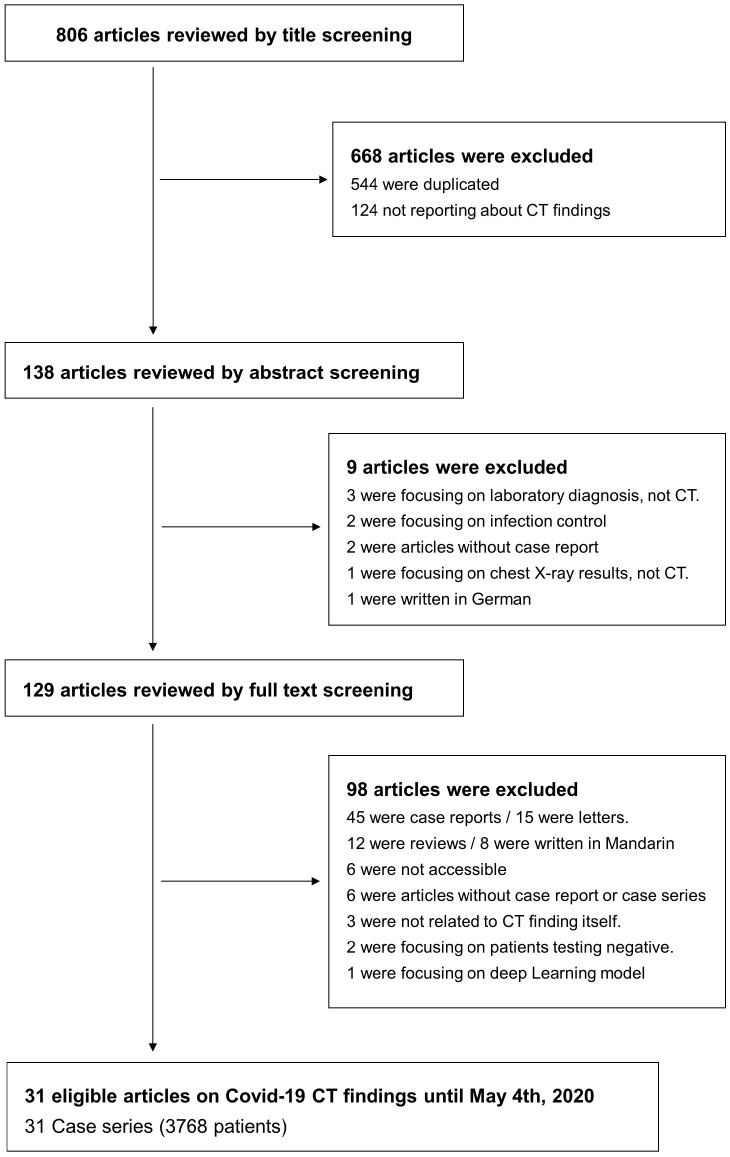
Flow chart of literature search.

Four investigators did the search and manually screened the data. HK, JS, and SL separately extracted data, and KHL double-checked to determine whether the eligible articles met the inclusion criteria. The final search was carried out on May 24, 2020, and excluded 544 overlapping or duplicated data sets. We first excluded duplicate articles and then labeled all the articles by examining titles, abstracts, and full texts in order. From 806 articles, a total of 31 case series with 3768 patients were included for the primary outcome. A flow-chart of literature search is presented in Figure 1.

### Analysis of included studies

In this current review, descriptions of the lungs of COVID-19 infected patients based on CT scans reported in the studies were accessed. Studies only describing saturation and chest X-ray findings were not included when the diagnosis was not mentioned, and no CT scan was performed. Each case series is described in Table S4-S5. We organized the data of the patients' characteristics, including age, sex, comorbidities, the period between symptom onset and admission/CT scan, clinical presentations, laboratory findings and types of treatments for analyses.

## Results

### Summary of previously published meta-analyses

A total of eleven meta-analyses on COVID-19 related to Chest CT findings have been published. Detailed description of each study is shown in Table 1. Three papers- Kim et al. 12, Xu et al 15 and Adams et al. 17 - summarized the sensitivity and specificity of chest CT as a diagnostic tool in COVID-19 pneumonia. However, there are no results about clinical presentations, laboratory findings, and treatment modalities. Four studies - Bao et al. 13, Zhu et al. 14, Sun et al. 20 and Lv et al. 21 - have conducted meta-analyses with only CT findings of COVID-19 patients, not describing clinical severity Only Wan et al. 18 summarized the both clinical characteristics and Chest CT findings of patients with COVID-19, but there is no results about laboratory findings and type of treatment. Other two papers - Park et al. 11 and Zheng et al. 16 - performed comparative analyses of CT findings comparing patients diagnosed in Wuhan with outside of Wuhan in China and the common patients with severe patients, respectively. Both studies contained no result about laboratory findings and treatment modalities. Chang et al. 18 summarized CT findings, clinical characteristics and outcomes only in small number of children without detailed laboratory findings or treatment in the manuscript.

### Baseline characteristics of patients included in case series

We included a total of 3768 patients with COVID-19 by compiling a total of 31 case series. Baseline characteristics are presented in Table 2. The mean age was 47.7 years. The percent ratio of males to females was approximately 48.5:51.7 (1826:1947). The most common comorbid conditions were hypertension (18.4%) and diabetes mellitus (8.3%). Cardio (7.1%) and cerebrovascular diseases (5.9%) were also common. The most frequent symptom was fever (78.7%), followed by cough (60.2%). Non-specific symptoms such as anorexia and fatigue or weakness were also highly prevalent, with their prevalence being 40.2% and 26.5%, respectively. Of the 17 types of initial symptoms examined, six were respiratory symptoms: cough, sputum, sore throat, nasal congestion or runny nose, shortness of breath, and hemoptysis. It took an average of 5.6 days from the onset of symptoms to hospital admission.

### Characteristics of CT findings in patients with COVID-19

Table 3 shows the characteristic CT findings in patients with COVID-19. The most common finding was vascular enlargement (84.8%), while the second most common feature was ground-glass opacity (GGO) (60.1%), followed by air-bronchogram (47.8%), and consolidation (41.4%). Other findings such as crazy paving, septal thickening, and pleural effusion accounted for a small percentage. Most lung lesions were located in the lung periphery (72.2%) and were present bilaterally (76%). Regarding lobe distribution, the right lower lobe (RLL) was the area where COVID-19 lesions most commonly occurred (72.2%), followed by the left lower lobe (LLL) (69.6%). Right upper and middle lobe (RUL, RML), and left upper lobe (LUL) were involved at a similar rate respectively (49.2, 49.5, 51.8 %).

### Laboratory data of patients with COVID-19

Average white blood cell (WBC) count was 5.20 ×10^9^/L and lymphocyte was 1.30 ×10^9^/L, indicating both were within normal range. More specifically, most patients showed a normal range of WBC counts (96.4%), while increased or decreased levels were shown in only 2.2% and 1.4% of patients, respectively. Similarly, lymphocytes were in normal range in most cases (87.2%). C-reactive protein was elevated in 31.7% of patients with a mean value of 13.7 mg/dL. Liver enzymes including aspartate transaminase (mean value: 36.93 U/L) and alanine aminotransferase (30.81 U/L) were within normal range, and lactic acid dehydrogenase (238.10 U/L) also showed normal level (Table 4).

### Treatment of patients with COVID-19

About 17.7% of patients received antiviral treatment, and 9.9% of patients had oxygen treatment. In the intensive care unit, 41 (1.1%), 4 (0.1%), and 2 (0.05%) patients received mechanical ventilator, extracorporeal membrane oxygenation (ECMO), and continuous renal replacement therapy (CRRT), respectively. Interestingly, plasminogen therapy was also tried in 13 patients with COVID-19 (Table 5).

## Discussion

Up to the best of our knowledge, there have been 11 meta-analyses about Chest CT findings of COVID-19 have been identified until now (Table 1). However, 4 papers 13, 14, 20, 21 simply described CT findings of patients with COVID-19. Another 3 meta-analyses 12, 15, 17 were only for sensitivity or specificity values of chest CT as a diagnostic tool of COVID-19. The other papers analyzed with partial sample sizes (comparative analyses between two groups 11, 16, children 18, ect.) Among these meta-analyses, there has been no comprehensive studies collecting all case series studies on data regarding the findings on chest CT in patients with confirmed COVID-19 and their association with the clinical severity, laboratory findings and treatment modalities of the viral infection.

Clinical deterioration by COVID-19 is characterized by ARDS due to acute lung injury, cytokine storm syndrome, and multiple organ failure. Among them, acute lung damage is the most representative clinical complication due to COVID-19 and is the starting point for all other serious clinical complications 22, 23. As already shown in previous epidemics, including severe acute respiratory syndrome (SARS) and middle east respiratory syndrome (MERS), the coronavirus enters the cell by using angiotensin-converting enzyme-2 receptor (ACE2R) 24. The ACE2Rs are mainly distributed in alveolar epithelial cells of the lungs, and thereby mediate direct lung injury by the coronavirus 25, 26. However, careful analysis of serious complications might offer a window of opportunity in the management of the condition. The radiographic information of a chest CT scan has the potential to provide valuable information and assist healthcare workers in predicting the acuity of the infection and in categorizing patients at low, medium, and high risk of mortality from the disease.

Most medical guidelines consider a chest CT scan as a useful diagnostic measure to complement for a RT-PCR test in spite of low specificity and unwanted exposure of radiation 8, 27, 28. Chest CT provides information to assess clinical severity and helps to predict clinical progress at the same time as diagnosis 29.

Pneumonia by various viruses, including novel coronavirus, shares radiologic characteristics that are different from bacterial pneumonia. Despite a few differences according to pathogenic mechanisms, viral pneumonia is commonly characterized by bilateral lung involvement and a GGO pattern that often show mixed appearance with consolidative lesions. In addition, viral pneumonia is mainly distributed in the lung periphery 30. Especially, in the COVID-19 pandemic, the radiologic characteristics of pneumonia caused by SARS and MERS coronavirus are something to be noted. The two diseases have shown that the mixed lesions occur mainly in the peripheral and lower regions of both lungs 8.

Several researchers have reported the radiologic characteristics of COVID-19 since the initial outbreak 31. Such information could provide important clues in understanding the pathogenic mechanism of SARS-CoV-2 and might play an important role in early diagnosis along with serologic testing 8, 31, even though the argument over the role of chest CT in COVID-19 diagnosis still persists 12, 28. In a study of 51 patients diagnosed with COVID-19, pneumonia mainly involved both lungs (86% of total patients / 97% of total lesions), and the lung lesions were distributed across multiple sites in more than 4 lobes (63% / 90%) 32. In addition, it occurred frequently in lower lobes (90% / 53%) and were mainly distributed in the posterior (80% / 89%) and peripheral areas of the lung (86% / 91%). Radiologic features of pneumonia from COVID-19 may depend on severity. A recent study divided COVID-19 into 5 stages (ultra-early, early, rapid progress, consolidation, and dissipation stages) and described the radiologic features of each stage 27. According to this study, single, double, or scattered GGO lesions were observed in the ultra-early stage, and during the early stage, the GGO patterns gradually expanded with the onset of interlobular interstitial edema. In the rapid progress stage, as inflammation intensified, the exudate in alveoli spread into nearby alveoli and interstitial spaces, and the GGO lesion progressed to consolidation with signs of air-bronchogram. In the consolidation stage, large scale patchy consolidation was clearly present. Lastly, in the dissipation stage, the existing consolidation was gradually absorbed and faded, the interlobular septum became relatively clear, and opacity of the reticular pattern was mainly observed.

Compared to 11 previous published meta-analyses, our study showed different the radiologic characteristics of chest CT in a total of 3768 COVID-19 patients by compiling case series studies. The most common feature of the CT findings of COVID-19 was vascular enlargement (or thickening) (Table 3). Although the finding is somewhat non-specific and the underlying mechanisms are unclear, a recent study indicated that it could be an important point that distinguishes COVID-19 pneumonia from non-COVID-19 pneumonia, and a useful indicator to suspect COVID-19 infection at an early stage 31, 33. The second most common CT finding in COVID-19 pneumonia was GGO, which has already been considered as a typical feature in viral pneumonia, including COVID-19. The GGO is a radiologic opacity that does not obscure the contours of close broncho-vascular structures, which suggests the existence of transudate or exudate in alveoli, but the fluid amount is not large enough to make a large contrast with the surrounding air spaces. Thus, the GGO pattern suggests early lesions such as alveolitis, and, as the disease progresses, it develops into a consolidative lesion by the accumulation of fibrinous exudate. In the GGO lesion, the alveoli are relatively better aerated compared to the severity of radiologic findings, such that asymptomatic COVID-19 infections are often found with significant abnormal findings in chest radiography. Another common CT finding for COVID-19 was air-bronchogram. This was reported at a similar rate as consolidation in our study because it has the same clinical significance as a secondary lesion following consolidation. Consolidation in the lung means that the functional space of alveoli has been lost by inflammatory exudates, thus predicting clinical exacerbation. Many studies, including this study, report mixed GGO and consolidation as a typical feature of COVID-19 34, and these mixed findings are understood as a process of transition in which the GGO lesions worsen into a consolidative stage rather than independent lesions. Other chest CT findings of COVID-19 included crazy paving, which is thought to be one of the worsening lesions of the GGOs that does not progress to consolidation. Based on the localization of chest CT findings of COVID-19 in this study, as in other existing studies, peripheral distribution is mainly shown, and the lesions usually involve the right lung and lower lobes. Although there is no clear evidence, these findings may be associated with the inflow of coronavirus particles through breathing. When the coronavirus particles enter the lung, it is easier to contact alveolar epithelial cells in the lung periphery where airflow slows down. Also, in regard to the preference for specific lung lobes in occurrence of COVID-19 pneumonia, we could understand such a tendency by referring to the volume of each lung segment. In a study of COPD patients, the assessment of the lung lobar volumes in the control group through three-dimensional (3D) CT showed that RLL had the largest volume and that the volumes got smaller in the order of LUL, LLL, RUL, and RML 35. Although these results do not exactly correspond to the localization of pneumonia from COVID-19 shown in this study, the preferred area for the occurrence of pneumonia seems to be associated with the physiologic lobar volumes.

A comprehensive diagnostic approach should be adopted for patients with known COVID-19, suspected cases, and for exposed individuals. The radiologic imaging, clinical picture, laboratory testing, and molecular confirmatory assays should be analyzed in a complementary and accumulative manner. Healthcare providers should consider all clinical cues before planning management and treatment.

## Conclusions

In this study, we provide information on the characteristics of CT findings of COVID-19 from a large number of patients through a comprehensive analysis of existing case series. The understanding of radiologic characteristics in COVID-19 can help assess and predict the clinical course of the disease, and provide a useful measure for early diagnosis of COVID-19. Above all, understanding the underlying mechanisms of radiologic patterns in COVID-19 is likely to provide a clue on the clinical properties of SARS-CoV-2 that causes serious lung damage with high infectivity. The findings and hallmarks in the chest CT could assist in predicting the severity and acuity, progression, prognosis, and risk of mortality of infection.

## Supplementary Material

Supplementary materials and tables.

## Author Contributions

JIS designed the study, KHL, HK, JS and SML collected the data and did the analysis. RAG, KHL, JSK, SL, SHH, KSK, KEK, AK, LJ, LS, HL, AK and JIS wrote the first draft of the manuscript and gave critical comments on manuscript draft. All authors had full access to all the study data. All authors reviewed wrote and approved the final version. The corresponding author had final responsibility for the decision to submit for publication.

## Figures and Tables

**Table 1:** Main characteristics and findings of the previous meta-analyses of chest CT in COVID-19

Authors	No. of studies	Sample size	Description	Comments
Park et al. 2020 11	9	627	The role of chest CT in COVID-19 diagnosis is inconclusive.	A comparative analysis of clinical presentations and CT findings was performed between patients diagnosed in Wuhan and outside of Wuhan in China - no significant findings about CT findings and no data about laboratory findings and type of treatment
Kim et al. 2020 12	68	6218	The pooled sensitivity was 94% (95% CI: 91-96; I2=95%) and specificity was 37% (26-50; 83%) for chest CT.	Summarized the sensitivity and specificity of chest CT as a diagnostic tool in COVID-19 pneumonia - there is no results about clinical presentations, laboratory findings, and treatment modalities
Bao et al. (2020) 13	13	2738	Typical CT signs were GGO (83.3%), GGO with mixed consolidation (58.4%). The incidences were highest in the right lower lobe (87.2%), left lower lobe (81.4%), and bilateral lower lobes (65.2%).	CT findings of patients with COVID-19 are summarized in this article - there is no results about clinical presentations, laboratory findings and type of treatment
Zhu et al. (2020) 14	7	4121	Most patients presented bilateral lung involvement (73.8%, 95% CI: 65.9-81.1) and the most common changes in lesion density were GGO (68.1, 56.9‐78.2).	CT findings of patients with COVID-19 are summarized in this article - not mentioning clinical presentations, laboratory findings, and type of treatment
Xu et al. (2020) 15	16	3186	Chest CT has a high sensitivity 92% (95% CI: 86-96), for detecting COVID-19, especially in a region with severe epidemic, which is helpful to early recognize suspicious cases and might contribute to confine epidemic.	Summarized the sensitivity of chest CT as a diagnostic tool in COVID-19 pneumonia - there is no results about clinical presentations, laboratory findings, and treatment modalities
Zeng et al. (2020) 16	15	2,451	Based on the CT images, the common patients were less frequent to exhibit consolidation (OR: 0.31), pleural effusion (0.19), lymphadenopathy (0.17), crazy-paving pattern (0.22), interlobular septal thickening (0.27), reticulation (0.20), traction bronchiectasis (0.40) with over 2 lobes nvolved (0.07) and central distribution (0.18) while more frequent to bear unilateral pneumonia (4.65) involving 1 lobe (13.84) or 2 lobes (6.95) when compared with severe patients.	The paper compared the common patients with severe patients - not comparing or mentioning about clinical presentations laboratory findings, and treatment modalities.
Adams et al. (2020) 17	6	1431	Chest CT appears to have a relatively high sensitivity (92.9% to 97.0%) in symptomatic patients at high risk of COVID-19, but it cannot exclude COVID-19. Specificity is poor (25.0% to 71.9%).	Summarized the sensitivity of chest CT as a diagnostic tool in COVID-19 pneumonia - there is no results clinical presentations, laboratory findings, and type of treatment
Chang et al. (2020) 18	9	93	In Fever occurred in 59% of the patients, while cough in 46%. Gastrointestinal symptoms (12%) were uncommon. There are 26% children are asymptomatic. The most common radiographic finding was GGO (48%).	The paper summarized CT findings, clinical characteristics and outcomes only in small number of children (not for adults) - detailed laboratory findings or treatment are not described in the manuscrupt
Wan et al. (2020) 19	14	1115	Chest CTs showed pure GGO (69%, 95% CI 58-80) and 70% (95% CI 46-95) of cases showed a location preference for the right lower lobe, 65% (58-73) of patients presented with ≥3 lobes involvement. In terms of clinical features, muscle soreness (21%, 95% CI 15-26) and diarrhea (7%, 4-10) were minor symptoms compared to fever (80%, 74-87) and cough (53%, 33-72).	CT findings and clinical presentations of patients with COVID-19 are summarized in this article - there is no results about laboratory findings and treatment modalities
Sun et al. 2020 20	55	NA	Pulmonary lesions more often involved bilateral lungs (78%, 95% CI: 45-100) and were more likely to have a peripheral (65.35%, 25.93-100). GGO (58.05%, 16.67-100), consolidation (44.18%, 1.61-71.46) and GGO plus consolidation (52.99%, 19.05-76.79) were the most common findings.	Investigation of CT findings of COVID-19 pneumonia- there is no findings about clinical presentations and laboratory findings
Lv et al. 2020 21	103	5673	The sensitivity in case series was 96% (95% CI: 0.93-0.99). The most common imaging manifestation was GGO which was found in 75% (0.68-0.82) of the patients. The pooled probability of bilateral involvement was 84% (0.81-0.88). The most commonly involved lobes were the right lower lobe (84%, 95% CI: 0.78-0.90) and left lower lobe (81%, 0.74-0.87).	CT findings of patients with COVID-19 are summarized in this article - there is no results about clinical presentations, laboratory findings and treatment modalities

CI: confidence interval; COVID-19: coronavirus disease 19; CT: computed tomography; GGO: Ground glass opacities; NA: not mentioned; No: Number.

**Table 2:** Patient characteristics of included case series

Variables	Mean or N/ total (%)	
Age (years)	47.7	
Sex (male/ female)	1826/ 1947	(48.5/ 51.7%)^ a^
**Underlying diseases**		
Hypertension	165/ 895	(18.4%)
Diabetes mellitus	65/ 782	(8.3%)
Cardiovascular disease	52/ 737	(7.1%)
Cerebrovascular disease	13/ 219	(5.9%)
Chronic liver disease	12/ 297	(4.0%)
Chronic kidney disease	8/ 281	(2.8%)
COPD	37/ 720	(5.1%)
Malignancy	24/ 417	(5.8%)
Pregnancy	2/ 62	(3.2%)
Others ^b^		
**Initial symptoms**		
Fever	2066/ 2624	(78.7%)
Cough	1438/ 2390	(60.2%)
Loss of appetite	74/ 184	(40.2%)
Fatigue or weakness	457/ 1727	(26.5%)
Sputum	425/ 1649	(25.8%)
Dyspnea	211/ 1043	(20.2%)
Myalgia	329/ 1937	(17.0%)
Sore throat	189/ 1330	(14.2%)
Chest tightness	71/ 635	(11.2%)
Chill	46/ 448	(10.3%)
Headache	147/ 1457	(10.1%)
Nasal congestion or runny nose	67/ 804	(8.3%)
Chest pain	35/ 487	(7.2%)
GI symptom	96/ 1523	(6.3%)
Shortness of breath	46/ 869	(5.3%)
Hemoptysis	13/ 651	(2.0%)
Others ^c^		
**Period from Sx onset to Adm (days) ^d^**	5.6	

Data are presented as mean value or the number of reported cases (N) compared to total cases.^ a^ In some included studies, inaccurate results were reported, so the sum does not match.^ b^ This contains diseases such as cerebral infarction, pulmonary emphysema, HIV infection, endocrine disease, surgical history and respiratory disease. ^c^ This contains symptoms such as lymphocytopenia, rhinobyon and snivel. ^d^ Total number of included cases was 351. Adm: admission; COPD: chronic obstructive pulmonary disease; GI: gastrointestinal; Sx: symptom.

**Table 3:** Chest CT findings of patients with COVID-19

CT findings	N/ total (%)	
**Imaging finding**		
Vascular enlargement	412/ 486	(84.8%)
GGO	1311/ 2182	(60.1%)
Air bronchogram	406/ 850	(47.8%)
Consolidation	811/ 1960	(41.4%)
Crazy paving	240/ 841	(28.5%)
Septal thickening	280/ 1317	(21.3%)
Pleural effusion	43/ 635	(6.8%)
**Transverse distribution**		
Central	51/ 264	(19.3%)
Peripheral	574/ 795	(72.2%)
Central and Peripheral	71/ 170	(41.8%)
**Lung region distribution**		
Unilateral	40/ 225	(17.8%)
Bilateral	1335/ 1757	(76.0%)
**Lobe distribution**		
RUL	147/ 299	(49.2%)
RML	148/ 299	(49.5%)
RLL	216/ 299	(72.2%)
LUL	155/ 299	(51.8%)
LLL	208/ 299	(69.6%)

Data are presented as the number of reported cases (N) compared to total cases. CT: computed tomography; COVID-19: coronavirus disease 2019; GGO: ground glass opacity; RUL: right upper lobe; RML: right middle lobe; RLL: right lower lobe; LUL: left upper lobe; LLL: left lower lobe.

**Table 4:** Laboratory findings of patients with COVID-19

Laboratoy findings	Mean or N/ total (%)	
WBC	5.20 ×10^9^/L^ a^	
Normal	1166/ 1210	(96.4%)
Increased	27/ 1210	(2.2%)
Decreased	17/ 1210	(1.4%)
Neutrophil	3.50 ×10^9^/L^ b^	
Lymphocyte	1.30 ×10^9^/L^ c^	
Normal	1130/ 1297	(87.2%)
Increased	55/ 1297	(4.2%)
Decreased	112/ 1297	(8.6%)
C-Reactive Protein	13.7 mg/dL^ d^	
Increased	376/ 1187	(31.7%)
Aspartate aminotransferase	36.9 U/L^ e^	
Alanine aminotransferase	30.8 U/L^ e^	
Lactic acid dehydrogenase	238.1 U/L^ f^	

Data are presented as mean value or the number of reported cases (N) compared to total cases. Total numbers of included cases are ^a^ 1171, ^b^ 1004, ^c^ 1329, ^d^ 1187, ^e^ 259, and ^f^ 1082. COVID-19: coronavirus disease 2019; WBC: white blood cell.

**Table 5:** Treatments of patients with COVID-19

Treatments	N (%)
Anti-coronavirus treatment	668 (17.7%)
Glucocorticoids	139 (3.7%)
Oxygen therapy	373 (9.9%)
Mechanical ventilator	41 (1.1%)
Plasminogen therapy	13 (0.3%)
ECMO	4 (0.1%)
CRRT	2 (0.05%)

Data are presented as the number of reported cases (N) compared to total cases (n=3768). COVID-19: coronavirus disease 2019; ECMO: extracorporeal membrane oxygenation; CRRT: continuous renal replacement therapy.
