# Supplementary material for: Chest CT abnormalities in COVID-19: a systematic review
Source: Int J Med Sci. 2021 Aug 1;18(15):3395–402. doi: 10.7150/ijms.50568 (PMC8436104; doi:10.7150/ijms.50568)
Supplement: Supplementary file 1 — Supplementary materials and tables. [file ijmsv18p3395s1.pdf]

**Supplementary Appendix**
**: “Chest CT abnormalities in COVID-19: a systematic review”**

**Table of contents**

1. PRISMA Checklist ..........................................................1
2. Search strategy ..........................................................3
3. Reasons for study exclusion..........................................................4
4. Detailed distribution of patients on the studies included studies..........................................................5

## 1. PRISMA Checklist

**Table S1. Checklist summarizing compliance with PRISMA guidelines***

| **Section/topic** | **#** | **Checklist item** | **Reported on page #** |
|---|---|---|---|
| **TITLE** | | | |
| Title | 1 | Identify the report as a systematic review, meta-analysis, or both. | Title |
| ABSTRACT | | | |
| Structured summary | 2 | Provide a structured summary including, as applicable: background; objectives; data sources; study eligibility criteria, participants, and interventions; study appraisal and synthesis methods; results; limitations; conclusions and implications of key findings; systematic review registration number. | 1 |
| INTRODUCTION | | | |
| Rationale | 3 | Describe the rationale for the review in the context of what is already known. | 2 |
| Objectives | 4 | Provide an explicit statement of questions being addressed with reference to participants, interventions, comparisons, outcomes, and study design (PICOS). | 2 |
| METHODS | | | |
| Protocol and registration | 5 | Indicate if a review protocol exists, if and where it can be accessed (e.g., Web address), and, if available, provide registration information including registration number. | N/A |
| Eligibility criteria | 6 | Specify study characteristics (e.g., PICOS, length of follow-up) and report characteristics (e.g., years considered, language, publication status) used as criteria for eligibility, giving rationale. | 2-3 |
| Information sources | 7 | Describe all information sources (e.g., databases with dates of coverage, contact with study authors to identify additional studies) in the search and date last searched. | 2-3 |
| Search | 8 | Present full electronic search strategy for at least one database, including any limits used, such that it could be repeated. | 2-3 |
| Study selection | 9 | State the process for selecting studies (i.e., screening, eligibility, included in systematic review, and, if applicable, included in the meta-analysis). | 2-3 (Figure1) |
| Data collection process | 10 | Describe method of data extraction from reports (e.g., piloted forms, independently, in duplicate) and any processes for obtaining and confirming data from investigators. | 2-3 |
| Data items | 11 | List and define all variables for which data were sought (e.g., PICOS, funding sources) and any assumptions and simplifications made. | 2-3 |
| Risk of bias in individual studies | 12 | Describe methods used for assessing risk of bias of individual studies (including specification of whether this was done at the study or outcome level), and how this information is to be used in any data synthesis. | N/A |
| Summary measures | 13 | State the principal summary measures (e.g., risk ratio, difference in means). | 2-3 |

| | | | |
|---|---|---|---|
| Synthesis of results | 14 | Describe the methods of handling data and combining results of studies, if done, including measures of consistency (e.g., $I^2$) for each meta-analysis. | 4 |

| Section/topic | # | Checklist item | Reported on page # |
|---|---|---|---|
| Risk of bias across studies | 15 | Specify any assessment of risk of bias that may affect the cumulative evidence (e.g., publication bias, selective reporting within studies). | N/A |
| Additional analyses | 16 | Describe methods of additional analyses (e.g., sensitivity or subgroup analyses, meta-regression), if done, indicating which were pre-specified. | N/A |
| RESULTS | | | |
| Study selection | 17 | Give numbers of studies screened, assessed for eligibility, and included in the review, with reasons for exclusions at each stage, ideally with a flow diagram. | 4-6 |
| Study characteristics | 18 | For each study, present characteristics for which data were extracted (e.g., study size, PICOS, follow-up period) and provide the citations. | 4-6, Table 2, Supplementary tables |
| Risk of bias within studies | 19 | Present data on risk of bias of each study and, if available, any outcome level assessment (see item 12). | Supplementary tables |
| Results of individual studies | 20 | For all outcomes considered (benefits or harms), present, for each study: (a) simple summary data for each intervention group (b) effect estimates and confidence intervals, ideally with a forest plot. | 5-8, Supplementary tables |
| Synthesis of results | 21 | Present results of each meta-analysis done, including confidence intervals and measures of consistency. | 5-8 |
| Risk of bias across studies | 22 | Present results of any assessment of risk of bias across studies (see Item 15). | N/A |
| Additional analysis | 23 | Give results of additional analyses, if done (e.g., sensitivity or subgroup analyses, meta-regression [see Item 16]). | N/A |
| DISCUSSION | | | |
| Summary of evidence | 24 | Summarize the main findings including the strength of evidence for each main outcome; consider their relevance to key groups (e.g., healthcare providers, users, and policy makers). | 8-9 |
| Limitations | 25 | Discuss limitations at study and outcome level (e.g., risk of bias), and at review-level (e.g., incomplete retrieval of identified research, reporting bias). | 9-10 |
| Conclusions | 26 | Provide a general interpretation of the results in the context of other evidence, and implications for future research. | 10 |
| FUNDING | | | |
| Funding | 27 | Describe sources of funding for the systematic review and other support (e.g., supply of data); role of funders for the systematic review. | 10 |

*Moher D, Liberati A, Tetzlaff J, Altman DG; PRISMA Group (2009) Preferred reporting items for systematic reviews and meta-analyses: the PRISMA statement. J Clin Epidemiol 62:1006-1012.

**2. Search strategy**

We searched PubMed, MEDLINE, Scopus, Embase and Web of Science for reports published only in English until May 4, 20200, that assessed the association between CT finding and all informations of patients with COVID-19. We searched all fields for coronavirus (search terms:"Coronavirus 19", "COVID-19", "SARS-CoV-2", "2019-nCoV", "novel coronavirus 2019") and terms of CT (search terms:"CT", "Computed Tomography").

Full search strategies for each database are given in Table S2.

**Table S2. Search strategy**

| Database | Number of studies | Search terrns |
|---|---|---|
| **PubMed** | 259 | ("Coronavirus 19" OR "COVID-19" OR "SARS-CoV-2" OR "2019-nCoV" OR "novel coronavirus 2019")<br>AND<br>("CT" OR "computed tomography") |
| **Medline** | 222 | ("Coronavirus 19" OR "COVID-19" OR "SARS-CoV-2" OR "2019-nCoV" OR "novel coronavirus 2019")<br>AND<br>("CT" OR "computed tomography") |
| **Scopus** | 85 | TITLE-ABS-KEY("Coronavirus 19" OR "COVID-19" OR "SARS-CoV-2" OR "2019-nCoV" OR "novel coronavirus 2019)<br>AND TITLE-ABS-KEY(CT OR "computed tomography") |
| **Embase** | 200 | ('Coronavirus 19' OR 'COVID-19' OR 'SARS-CoV-2' OR '2019-nCoV' OR 'novel coronavirus 2019')<br>AND<br>(CT OR 'computed tomography') |
| **Web of Science** | 40 | ((TS=Coronavirus 19) OR (TS=COVID-19) OR (TS=SARS-CoV-2) OR (TS=2019-nCoV) OR (TS=novel coronavirus 2019))<br>AND<br>((TS=CT)OR (TS=computed tomography)) |

**3. Reasons for study exclusion**

We manually screened the retrieved articles which were met inclusion criteria. After excluding studies by examining titles and abstracts, full texts of 31 studies were eligible for inclusion. 98 studies were retrieved following reasons**:**

**Table S3: Reason for exclusion during full text screening**

| Number of studies | Reason |
|---|---|
| **45** | Case reports |
| **15** | Letters |
| **12** | Reviews |
| **8** | Written in Mandarin |
| **6** | Not accessible |
| **6** | Articles without case report or case series |
| **3** | Not related to CT finding itself |
| **2** | Focusing on patients testing negative for COVID-19 |
| **1** | Focusing on Deep Learning model |

CT: computed tomography, COVID-19: Coronavirus disease 19

## 4. Detailed description of included studies

### Table S4: Detailed description about basal characteristics of included case-series

| No. | Authors | Sample size | Mean Age | Sex (M:F) | Initial symptoms | | Period between symptom onset and admission (day) | Period between symptom onset (or admission) and CT scan (day) | CT image feature | | | | |
|---|---|---|---|---|---|---|---|---|---|---|---|---|---|
| | | | | | Fever | Other | | | Distribution | Lobes | Appearance | Specific signs | CT score* |
| 1 | Zhao D et al. (2020 March) [1] | 19 | 48 (IQR:27 ~56) | 11:8 | 15/19 (78.95%). | Cough 9/19 (47.37%) sore throat 4/19 (21.05%) headache 2/19 (10.53%), fatigue 2/19 (10.53%), Diarrhea 1/19 (5.26%), Chest tightness 1/19 (5.26%) | 5 (IQR: 3~9) | 3.76±2.22 (1-10) // 5.2±1.2 (4-8) times | - | N/A | Multiple mottling and GGO 17/19(89.47%) | N/A | N/A |
| 2 | Caruso. D et al. (2020 April) [2] | 158 | 57 ±17 | 83:75 | 97/158 (61%) | Cough 88/158 (56%), dyspnea 52/158 (33%), lymphocytopenia 95/158 (60%) | N/A | N/A | Right upper lobe 53/58(91%), Right middle lobe 48/58(82%), Right lower lobe 54/58(93%) Left upper lobe 49/58(84%) Left lower lobe 53/58(91%) | N : 4.41±2.26 | GGO 58/58 (100%), Consolidation 42/58 (72%), | Crazy-paving: 23/58 (39%) Rounded morphology: 19/58(32%) Linear oppacities16/58(27%) Air bronchogram 21/58(36%) Interlobular septal thickening 8/58(13%) | N/A |
| 3 | A Bernheim et al. (2020 Feb) [3] | 121 | 45±15. 6 (18-80) | 61:60 | 74/121 (61%) | Cough 58/121 (48%), sputum production 20/121(17%) | N/A | Early (0-2days): 36/121(29.7%) intermediate (3-5days): 33/121(27.2%) Late: 25/121(20.6%) | Right upper lobe 53/121(44%), Right middle lobe 50/121(41%), Right lower lobe 79/121(65%) Left upper lobe 58/121(48%) Left lower lobe 76/121(63%) | - | - | Crazy-paving: 6/121 (5%) Rounded morphology: 65/121(54%) Linear oppacities 9/121(7%) Bronchial wall thickening 14/121(12%) | N/A |
| 4 | J Wu et al. (2020 Feb) [4] | 80 | 44±11 | 38:42 | 18/21 (86%). Low (<38) 19/80 (24%), Moderate (38.1-39) 38/80 (47%), High (>39.1) 3/80 (4%) | Cough 58/80 (73%), Expectortation 11/80(14%), Chest pain 5/80 (6%), Muscle ache 13/80(16%) Dyspnea 7/80(9%) Abdominal pain& diarrhea 7/80(9%) | 7±4 | N/A | Subpleural distribution 42 (53%) Diffuse distribution 7 (9%) Peribronchial distribution 3 (4%) Mixed distribution 24 (30%) | Average lung segments involved 12 (6) Dorsal segment of the right lower lobe 69 (86%) Lateral basal segment of the right lower lobe 64 (80%) Posterior basal segment of the right lower lobe 68 (85%) Dorsal segment of the left lower lobe 61 (76%) Posterior basal segment of the left lower lobe 65 (81%) PII value 34% (20%) | GGO 73/80 (91%) Consolidation 50 (63%) | Crazy paving pattern 23 (29%) Spider web sign 20 (25%) Subpleural line 16 (20%) Bronchial wall thickening 9 (11%) Lymph node enlargement 3 (4%) Pericardial effusion 4 (5%) Pleural effusion 5 (6%) | 4.3 (1-11) |
| 5 | YH Xu et al. (2020 Apr 17) [5] | 50 | 40.0±1 0 (27-60) | 29:21 | 37.3–38 °C (22 or 44%) 38.1–39 °C (16 or 32%) >39 °C (5 or 10%) | Cough 20 (40%), Expectoration7 (14%), headache 5(10%) Fatigue 8 (16%), Muscle ache 8 (16%) Chest tightness and dyspnea 4 (8%) Gastrointestinal reaction 1 (2%) | - | 4 (1-7) | - | - | GGO (76.5%), consolidation (11%) | Enlarged pulmonary vessels (70.6%), adjacent pleura thickening (41.2%), crazy paving (35.3%), air brochograms (29.4%), interlobar fissure (23.5%) | 4.3 (1-11) |
| 6 | Tao Ai et al. (2020 ) [6] | 1049 | 51±15 (2-95) | 467:582 | - | - | - | - | Consistent with viral pneumonia = 888 (88%) | - | GGO 409/888 (46%), consolidation 447/888 (50%) | Reticulation/thickened interlobular septa 8/888 (1%), nodular lesions 24/888 (3%) | |
| 7 | Shuchang Zhou et al. (2020 Feb 19) [7] | 62 | 52.8±1 2.2 (30-77) | 39:23 | 54/62 | coughing and sputum 28/62, fatigue 14/62, shortness of breath 15/62, muscle pain 20/62, abdominal pain or diarrhea 9/62 | - | | Peripheral 48 (77.4%), peripheral and central 14 (22.6%) | - | GGO 25/62 (40.3%), consolidation 21/62 (33.9%) | Air bronchogram 45/62 (72.6%), thickening of pleura 30/62 (48.4%), pleural effusion 6/62 (9.7%) | left 5.9±5.1, right 6.2 ± 5.3, upper 3.0±3.4, middle 4.5±3.8, lower 4.5±3.7, anterior 4.4±4.1, posterior 7.7±6.3 |
| 8 | Rui Zhang et al. (2020 Apr 1) [8] | 120 | 45.4±1 5.6 | 43:77 | 81 (68%) | Cough 74 (63%), dyspnea 38 (32%), myalgia or fatigue 57 (48%), headache 28 (23%), | - | | Bilateral 68 (57%), peripheral 109 (91%), central 39 (33%) | Upper right lobe 41 (34%), middle right lobe 50 (42%), lower right lobe 83 (69%), upper left lobe 48 (40%), lower left lobe 79 (66%) | GGO 111 (93%), consolidation 66 (55%) | Crazy paving 30 (25%), bronchiectasis 14 (12%), effusion 9 (8%) | - |

| | | | | | | | | | | | | | |
|---|---|---|---|---|---|---|---|---|---|---|---|---|---|
| **9** | **Kunwei Li et al. (2020 Mar 16) [9]** | 78 | 44.6±1 7.9 | 38:40 | 54 (69.2%) | Chills 5 (6.4%), cough 36 (46.2%), sputum 16 (20.5%), hemoptysis 2 (2.6%), sore throat 8 (10.3%), nasal congestion and runny nose 10 (12.8%), headache and dizziness 6 (7.7%) | 3±2 (0-15) | | Bilateral 45 (57.7%), peripheral 49/56 (87.5%) | More than two lung lobes 40 (51.3%) | GGO 45/56 (80.4%), consolidation 12/56 (21.4%) | Interlobular septal thickening 25/56 (44.6%), air brochogram 41/56 (73.2%), | - |
| **10** | **Michael Chung et al. (2020) [10]** | 21 | 51±14 (29-77) | 13:8 | 14/21 (67%) | Fatigue 3 (14%), headache 3 (14%), cough 9 (43%), muscle soreness 3 (14%), nausea 1 (5%) | - | | Bilateral 16 (76%) | More than two lung lobes 15 (71%) | GGO 18/21 (86%), consolidation 6 (29%) | Crazy paving pattern 4 (19%), peripheral distribution 7 (33%) | - |
| **11** | **Kai-Cai Liu et al. (2020 Mar 7) [11]** | 73. | 41.6±1 4.5 (5-86) | 41:32 | 68/73 (93%) | Cough 60 (82%), fatigue 55 (75%), sputum 39 (53%), anorexia 20 (27%) | - | | Unilateral 15/73 (20%), bilateral 55/73 (75%) | - | GGO 65/73 (89%), consolidation 8/73 (10%) | Paving stone sign 28 (38%), bronchial wall thickening 19 (26%) | - |
| **12** | **Chunqin Long et al. (2020 Mar 11) [12]** | 36 | 44.8±1 8.2 | 20:16 | 36/36 (100%) | Cough 27/36 (75%), myalgia or fatigue 14/36 (38.9%), nausea or diarrhea 6/36 (16.6%) | - | | Peripheral distribution 26/36 (72.2%), central distribution 10 (27.8%) | Multiple CT abnormalities 25/36 (69.4%) | GGO 11/36 (30.6%), consolidation 6/36 (16.7%), GGO with consolidation 19/36 (52.7%) | Lymphadenopathy 1/36 (2.78%), pleural effusion 2/36 (5.56%) | - |
| **13** | **Yuki Himoto et al. (2020 Mar 18) [13]** | 6 | 58.5 (45-81) | 5:1 | - | - | 9.5 (4-25) | | Peripheral predominance 6/6 (100%), bilateral 6/6 (100%) | - | GGO 4/6 (66%), GGO with consolidation 2/6 (33%) | Pulmonary nodules 2/6 (33.3%) | - |
| **14** | **Rui Han et al. (2020 Feb 15) [14]** | 108 | 45 (21-90) | 38:70 | 94/108 (87%) | Dry cough 65 (60%), fatigue 42 (39%), chest distress 17 (16%), pharyngeal pain 14 (13%), headache 14 (13%), muscle pain 12 (11%) | - | median 1 (1-3) | Peripheral 97 (90%), central 2 (2%), peripheral and central 9 (8%) | - | GGO 65 (60%), consolidation 6 (6%), GGO with consolidation 44 (41%) | Vascular thickening 86 (80%), crazy paving pattern 43 (40%), air bronchogram sign 52 (48%), halo sign 69 (64%) | - |
| **15** | **Xiaoli Zhang et al. (2020 Mar 15) [15]** | 573 | 46.65± 13.82 | 295:278 | 492/573 (85.9%) | Cough 392/573 (68.4%), expectoration 208 (36.3%), hemoptysis 11 (2%), sore throat 80 (14%), nasal obstruction 29 (5.1%), muscle ache 66 (11.5%), fatigue 109 (19%), shortness of breath 26 (4.5%), diarrhea 45 (7.9%), nausea and vomiting 22 (3.8%), headache 65 (11.3%) | - | | Bilateral 432 /573 (75.3%) | More than 2 lobes affected 230/573 (40.1%) | GGO or consolidation 573 (100%) | - | - |
| **16** | **Xiaoli Zhang et al. (2020 Mar 15) [15]** | 72 | 34.9±1 4.20 | 33:39 | 48/72 (66.7%) | Cough 33/72 (45.8%), expectoration 17 (23.6%), sore throat 17 (23.6%), nasal obstruction 7 (9.7%), muscle ache 5 (7.0%), fatigue 9 (12.5%), diarrhea 8 (11.1%), headache 2 (2.8%), | - | - | - | - | Absence of both GGO and consolidation 72 (100%) | - | - |
| **17** | **Hui Dai et al. (2020 Apr 1) [16]** | 234 | 44.6±1 4.8 (7-82) | 136:98 | 170 (72.6%) | pharyngeal discomfort (15%), fatigue (13.2%), chill (9.8%), muscle ache (9.0%), rhinobyon and snivel (5.6%), diarrhea (3.8%), chest pain (3.4%), chest tightness (5.6%), short of breath (2.1%), difficulty breathing (3%), nausea and vomiting (2.1%) | - | - | Bilateral multiple lung lobes 192/219 (87.6%), periphery and/or lower lungs 208/219 (94.98%) | - | - | VES 207/219, interlobular septal thickening 205/219, air bronchus sign 184/219, intralesional and/or perilesional bronchiectasis 173/219, pleural thickening 170/219, solid nodules 138/219, reticular/mosaic sign 135/219 | - |
| **18** | **Xi Xu et al. (2020 Feb 28) [17]** | 90 | 50 (18-86) | 39:51 | 70 (78%) | Cough 57 (63%), sputum 11 (12%), fatigue 19 (21%), myalgia 25 (28%), sore throat 23 (26%), chills 6 (7%), headache 4 (4%), diarrhea 5 (6%) | - | - | Periphery 46 (51%), bilateral 53 (59%), multifocal 62 (69%) | More than two lobes 53 (59%) | GGO 65 (72%), consolidation 12 (13%) | Crazy paving pattern 11 (12%), interlobular septal thickening 33 (37%), linear opacities combined 55 (61%), air bronchogram sign 7 (8%), adjacent pleura effusion 4 (4%) | - |
| **19** | **Chun Shuang Guan et al. (2020 Mar 6) [18]** | 53 | 42 (1-86) | 25:28 | - | - | - | - | Bilateral 37/47 (78.72%), subpleural distribution 44/47 (93.6%) | - | GGO 47 (100%), consolidation 30 (63.8%) | Crazy-paving 42 (89.3%), air bronchogram 36 (76.6%), | - |
| **20** | **K. Wang et al. (2020 Mar 4) [19]** | 114 | 53 (23-78) | 58:56 | 107/114 (93.9%) | Cough 91/114 (79.8%), sputum 9 (7.9%), sore throat 6 (5.3%), chest tightness 27 (23.7%), dyspnea 27 (23.7%), diarrhea 3 (2.7%) | - | - | Peripheral 48 (43.6%), bilateral 62 (56.4%) | - | GGO 30/114 (27.3%), consolidation 30 (27.3%), GGO with consolidation 50 (45.4%) | Pleural effusion 1 /114 (0.9%) | |
| **21** | **Wanbo Zhu et al. (2020 Mar 10) [20]** | 116 | 40 (27-53) | 56:65 | 84/116 (72%) | Cough 73/116 (63%), myalgia or fatigue 11 (9%), expectoration 22 (19%), chest stuffiness 5 (4%) | - | - | Bilateral 29/32 (91%) | - | GGO 15/32 (47%), consolidation 4 (13%) | Crazy-paving pattern 1/32 (3%), pleural effusion 2/32 (6%) | |
| **22** | **Pan F et al. (2020 Feb 13) [21]** | 21 | 40±9 (25-63) | 6:15 | Fever 84/116 (72%) | Throat pain 4/21 (19%), Cough 12/21 (57%), Expectoration 6/21 (29%), Chills 6/21 (29%), Fatigue 11/21 (52%), Loss of appetite 9/21 (43%), Myalgia 5/21 (24%), Chest pain 2/21 (9.5%) | 2±2 (0-9) | 2±2 (0-9) / Mean interval 4±1 (1-8), 4±1 (3-6) times | Single lobe 3/21 (14%), Bilateral Multilobe 18/21 (86%); Peripheral 13/21 (62%), Random | N : 2±2 (0-5) | GGO 15/21 (71%), Consolidation 19/21 (91%), | Crazy-paving: 4/21 (19%) | Total 2±2 (0-6). LUL 0±1 (0-2), LLL 1±1 (0-3), RUL 0±1 |

| | | | | | | | | | | | | | |
|---|---|---|---|---|---|---|---|---|---|---|---|---|---|
| | | | | | | | | | 7/21 (33%), Diffuse 1/21 (4.8%) | | | | (0-2), RML 0±1 (0-2), RLL 1±1 (0-2) |
| **23** | **Huang G et al. (2020 Mar 30) [22]** | 25 | 46.9±15.8 (20-81) | 14:11 | 18/21 (86%). Low (<38) 6/21 (29%), Moderate (38.1-39) 7/21 (33%), High (>39.1) 5/21 (24%) | Thorat pain 2/25 (8%), Cough 18/25 (72%), Expectoration 5/25 (20%), Chills 6/25 (24%), Fatiuge 10/25 (40%), Loss of apetite 10/25 (40%), Myalgia 3/25 (12%), Chest pain 2/25 (8%) | Onset to diagnosis and treatment : 3.76±2.22 (1-10) | 3.76±2.22 (1-10) // 5.2±1.2 (4-8) times | - | - | - | - | - |
| **24** | **Wang D et al. (2020 Mar 17) [23]** | 138 | 56 (IQR 42-68) | 75:63 | 21/25 (84%). Range 37.3-39.1 | Pharyngyalgia 24/138 (17.4%), Dry cough 82/138 (59.4%), Expectoration 37/138 (26.8%), Fatigue 96/138 (69.6%), Anorexia 55/138 (39.9%), Myalgia 48/138 (34.8%), Dyspnea 43/138 (31.2%), Diarrhea 14/138 (10.1%), Nausea 14/138 (10.1%), Dizziness 13/138 (9.4%), Headache 9/138 (6.5%), Vomiting 5/138 (3.6%), Abdominal pain 3/138 (2.2%) | Median 7.0 (IQR 4.0-8.0) | | Bilateral 138/138 (100%) | - | - | - | - |
| **25** | **Xiaoyu Han et al. (2020 Apr 17) [24]** | 17 | 40.0±10 (27-60) | 6:11 | 136/138 (98.6%). | Cough 7/17 (41%), weakness 2/17 (11%), sputum 2/17 (11%), headache 2/17(11%) | - | 4 (1-7) | Unilateral (29.4%), bilateral (70.6%) | - | GGO (76.5%), consolidation (11%) | Enlarged pulmonary vessels (70.6%), adjacent pleura thickening (41.2%), crazy paving (35.3%), air brochograms (29.4%), interlobar fissure (23.5%) | 4.3 (1-11) |
| **26** | **Shen Q et al. (2020) [25]** | 9 | 8 (1-12) | 3:6 | 14/17 (82.4%) among which 12 > 38 (85.7%) | Sore throat 1/9 (11%), Cough 1/9 (11%), Diarrhea 2/9 (22%) | Median 3 (0-17) | | | Unilateral 2/2 | GGO 2/9 (22%) | None | |
| **27** | **Wu Y et al. (2020) [26]** | 13 | 48 (30-78) | 10:3 | 4/9 (44.4%). Mean 37.3, Range 36.5-39.1 | - | - | - | - | - | - | - | - |
| **28** | **Gao L et al. (2020) [27]** | 6 | 40±10 | 1:5 | - | cough, sore throat, fatigue | | all within 2 days | Single lesion 2/6, multiple lesion 4/6 Subpleural 2/6, peribronchial 1/6 | Unilateral 4/6, bilateral 2/6 | GGO 5/6 | Lobar septal thickening 1/6, bronchial wall thickening 2/6, halo sign 2/6, crazy-paving 1/6, tree-in-bud 1/6 | |
| **29** | **Shi H et al. (2020) [28]** | 81 | 49.5±11 | 42:39 | fever | Dyspnea 34/81(42%), chest tightness 18 (22%), cough 48 (59%), sputum 15 (19%), rhinorrhea 21 (26%), weakness 7 (9%), headache 5 (6%), vomiting 4, diarrhea 3, dizziness 2, anorexia 1 | | | Bilateral 64 (79%), peripheral 44 (54%), ill-defined 66 (81%) | Involved lung segments: 10.5±6.4. | GGO 53 (65%) -> mainly RLL (27%) | | |
| **30** | **Zhao W et al. (2020 Feb) [29]** | 101 | 44.44±12.32 (17-75) | 56:45 | 59/81 (73%) : <37.3 (30%), 37.3-38 (25%), 38-39 (28%), >39 (17%) | cough 63 (62.4%), myalgia/fatigue 17(16.8%), sore throat 12 (11.9%), dyspnea 1, diarrhea 3, nausea/vomiting 2 | | admission to CT : 1 (0-7) | Peripheral 87.1%; Upper lung dominant 5.9%, lower lung dominant 54.5%, no craniocaudal 31.7%; Bilateral 82.2%, unilateral 9.9%; Focal 5.9%, multifocal 54.5%, diffuse 31.7% | | GGO 87 (86.1%), consolidation 44 (43.6%) | Architectural distortion (21.8%), bronchial wall thickening (28.7%), reticulation 48.5%, subpleural bands 27.7%, traction bronchiectasis 52.5%, pleural effusion 13.9%, vascular enlargement 71.3% | Extent score 6/39 (0-20) |
| **31** | **Zhou Y et al. (2020 Mar) [30]** | 17 | (18-70) | 6:11 | 79/101(78.2%) | Cough 8 (47.1%), dyspnea 2 (11.8%), fatigue 3 (17.6%), diarrhea 0 | | - | Bilateral 8 (47.1%), single lobe 4 (23.5%), normal 5 (41.7%) | - | - | - | - |
| **32** | **Wang Y et al. (2020) [31]** | 90 | 45±14 | 33:57 | 11/17 (64.7%) | Cough 20 (22%), Hypodynamia 22 (24%), Chest tightness 8 (9%), Sore throat 7 (8%), Diarrhea 6 (7%), Headache 4 (4%), Muscle pain 4 (4%) | | - | - | - | GGO 35/78(45%)-49/79(62%) | - | - |

Abbreviation : No. : Number; CT : computer tomography; GGO : ground glass opacity; WBC : white blood cell; CRP : C-reactive protein; ESR : erythrocyte sedimentation rate; N/A : not available

* The degree of involvement in each lung zone was scored

## Table S5: Detailed description about basal characteristics of included case-series

| No. | Authors | Initial symptoms | | | | Underlying disease | Clinical outcome / Course / Therapy | | | Comments |
|---|---|---|---|---|---|---|---|---|---|---|
| | | CBC | CRP/ESR (mg/dL) | Other inflammatory biomarkers | Blood chemistry | | Clinical outcome / Course | Therapy | Complications | |
| 1 | Zhao D et al. (2020 March) [1] | WBC 4.92 (1.26-7.63), ratio of neutrophil 74.02 (55.30-93), Lymphocyte 0.97 (0.3-2.03) | CRP 26.47 (10-127.1) | IL-6 19.34 (8.7-45.3) | AST 34.9(17.6-103.8) ALT 36.37(11.8-85.0) | N/A | N/A | N/A | N/A | Admission data was recruited 19 NCOVID-19 patients and 15 NON-COVID-19 patients from Jan 23 to Feb 5, 2020, at the Second Affiliated Hospital of Anhui Medical University and Suzhou Municipal Hospital in Anhui province, China 23 to Feb 5, 2020. |
| 2 | Caruso. D et al. (2020 April) [2] | Lymphocyte 1.08±0.47 | CRP (13.64 ± 38.68) | N/A | LDH 339.5±124.153 | N/A | N/A | N/A | N/A | CT feature was classified only in RT-PCR confirmed patients<br>Other features were about both RT-PCR confirmed patients and not confirmed patients (There were no Raw data) |
| 3 | A Bernheim et al. (2020 Feb) [3] | N/A | N/A | N/A | N/A | N/A | N/A | N/A | N/A | CT Feature were classified according to the time of admission |
| 4 | J Wu et al. (2020 Feb) [4] | WBC 5.40 (4.20–6.95) Neutrophil 3.74 (2.67–5.20); lymphocyte 1.15 (0.76–1.40) | CRP 12.39 (2.71–50.61) | N/A | N/A | COPD 1/17 (5%), HTN (5%), type 2 DM 2/17 (11%) | N/A | N/A | N/A | N/A |
| 5 | YH Xu et al. (2020 Apr 17) [5] | leukocyte 4.6±1.9, lymphocyte 1.2±0.4 | CRP 11.7 (11.8), ESR 34 (16) *(interquartile range) | N/A | AST 33 (24.5), 31 (22) (interquartile range) | N/A | mean treatment time : 22 (18-31) | anti-viral drug, empirical antibacterial drug, abidol hydrochloride | N/A | - |
| 6 | Tao Ai et al. (2020 ) [6] | - | - | - | - | N/A | - | - | - | From Jan 6 to Feb 6, 2020, a total of 1049 patients who were suspected of novel coronavirus infection and underwent both chest CT imaging and laboratory virus nucleic acid test were retrospectively enrolled in Tongji Hospital of Tongji Medical College of Huazhong University of Science and Technology, Wuhan, Hubeim China |
| 7 | Shuchang Zhou et al. (2020 Feb 19) [7] | leukopenia 6/30, decreased lymphocyte count 24/30, | increased hs-CRP 27/27, increased ESR 1/27 | - | - | HTN 4/62, DM 4/62, cerebral infarction 1/62, CKD 1/62, pregnancy 2/62 | - | - | - | A retrospective study of 62 consecutive patients with laboratory-confirmed COVID-19 pneumonia was performed. |
| 8 | Rui Zhang et al. (2020 Apr 1) [8] | WBC 5.0±2.2, neutrophil 2.0±1.7, lymphocyte 2.4±1.8 | - | - | LDH 235.6±109.6 | Diabetes 7 (6%), HTN 19 (16%), CVD 9 (8%), COPD 4 (3%), Malignancy 7 (6%) | - | - | - | this was a retrospective analysis of the clinical and thoracic CT features of 120 consecutive patients with confirmed SARS-CoV-2 pneumonia admitted to a tertiary university hospital between January 10 and February 10, 2020, in Wuhan city, China |
| 9 | Kunwei Li et al. (2020 Mar 16) [9] | - | - | - | - | HTN 10 (12.8%), DM 4 (5.1%), chronic liver disease 1 (1.3%), COPD 9 (11.5%), heart disease 2 (2.6%), tumor 3 (3.8%) | - | - | - | The authors conducted a retrospective single-center study on patients with COVID-10 from Jan. 18, 2020 to Feb 7, 2020 in Zhuhai, China. |
| 10 | Michael Chung et al. (2020) [10] | - | - | - | - | - | - | - | - | From January 18, 2020, until January 27, 2020, 21 patients admitted to three hospitals in three provinces in China with confirmed 2019-nCoV underwent chest CT. Ten patients were from Zhuhai (Guangdong Province) and were imaged with 1-mm-thick slices with a UCT 760 scanner (United Imaging, Shanghai, China). Nine patients were from Nanchang (Jiangxi Province) and were imaged with 8-mm-thick slices with an Emotion 16 scanner (Siemens Healthineers, Erlangen, Germany). Two patients were from Qingdao (Shandong Province) and were imaged with 5-mm-thick slices, one with a Bright Speed scanner (GE Medical Systems, Milwaukee, Wis) and one with an Aquilion ONE scanner (Toshiba Medical Systems, Tokyo, Japan). |
| 11 | Kai-Cai Liu et al. (2020 Mar 7) [11] | - | - | - | - | - | - | - | - | The data of patients were collected from 6 hospitals in Anhui province, China from Jan 21 to Feb 3, 2020. |
| 12 | Chunqin Long et al. (2020 Mar 11) [12] | Leukocyte count (normal or decreased) 33/36 (91.7%), decreased lymphocytes 23/36 (63.8%) | Leukocyte count (normal or decreased) 33/36 (91.7%), decreased lymphocytes 23/36 (63.8%) | - | - | - | - | - | - | From January 20th, 2019 to February 8th, 2020, a total of 204 patients suspected for COVID-19 underwent chest CT examinations. Of the patients, 106 were not tested using rRT-PCR. Eleven other patients were transferred to other hospitals and were also excluded. The remaining 87 patients underwent both CT and rRT-PCR in our hospital. The gold standard fora final diagnosis was positivity of first or repeated rRT-PCR tests. Amongst the 87 included cases, 36 patients were finally diagnosed with COVID-19 pneumonia. The other 51 patients without COVID-19 pneumonia served as the control group |

| | | | | | | | | | | |
|---|---|---|---|---|---|---|---|---|---|---|
| 13 | **Yuki Himoto et al. (2020 Mar 18) [13]** | - | - | - | - | Pulmonary emphysema 1/6 (16.6%) | - | - | - | This retrospective study included 21 patients clinically suspected COVID-19 pneumonia and underwent chest CT more than 3 days after the symptom onset: six patients confirmed COVID-19 pneumonia by real-time RT-PCR and 15 patients proved uninfected. |
| 14 | **Rui Han et al. (2020 Feb 15) [14]** | Decreased WBC count 11 (10%), decreased lymphocte count 65 (60%) | High CRP 107 (99%) | - | - | - | - | - | - | This retrospective study received local ethics committee approval. Patients with confirmed COVID-19 pneumonia confirmed by SARS-CoV-2 nucleic acid test (reverse transcription–polymerase chain reaction) at our hospital ( Wuhan No. 1 Hospital) from January 4 to February 3, 2020, were enrolled in this retrospective study |
| 15 | **Xiaoli Zhang et al. (2020 Mar 15) [15]** | leucocytes 5.01±1.87, neutrophils 3.29±1.68, lymphocytes 1.23±0.52 | CRP 8.8 (3.1-22.0) | Procalcitonin 0.05 (0.04-0.08) | LDH 213.0 (173-268), CK 73 (48-111), Glc 6.62±2.91, serum Cr 69.17±24.52, BUN 4.04±1.69, TB 11.26±8.04 | - | ARDS 14 (2.4%), shock 2 (0.3%), liver function abnormality 75 (13.1%), AKI 2 (0.3%) | Anti-coronavirus treatment 488 (85.2%), glucocorticoids 77 (13.4%), oxygen therapy 250 (43.6%), mechanical ventilation 9 (1.6%) | - | Patients confirmed with SARS-CoV-2 infection of Zhejiang province from Jan 17 to Feb 8 underwent CT or x-ray were enrolled. Epidemiological, clinical data were analyzed between those with abnormal or normal imaging findings. This group had abnormal imaging findings. |
| 16 | **Xiaoli Zhang et al. (2020 Mar 15) [15]** | leucocytes 5.42±2.00, neutrophils 3.48±1.88, lymphocytes 1.39±0.61 | CRP 2.3 (0.9-9.5) | Procalcitonin 0.05 (0.04-0.07) | LDH 174.5 (148-235.5), CK 62.5 (47-83.75), Glc 6.57±3.24, serum Cr 65.54±13.16, BUN 3.90±1.13, TB 9.11±4.86 | - | Liver function abnormality 6 (8.3%) | Anti-coronavirus treatment 56 (77.8%), oxygen therapy 8 (11.1%) | - | Patients confirmed with SARS-CoV-2 infection of Zhejiang province from Jan 17 to Feb 8 underwent CT or x-ray were enrolled. Epidemiological, clinical data were analyzed between those with abnormal or normal imaging findings. This group had normal imaging findings. |
| 17 | **Hui Dai et al. (2020 Apr 1) [16]** | - | - | - | | - | - | - | - | This was a multi-centered study included 234 inpatients from 13 hospitals during 113<br>17 days (from January 10th to February 7th 2020) in Jiangsu. All the cases were confirmed with 114<br>the criteria for SARS-CoV-2 infection established by National Health Commission, which was 115<br>consistent with one of the following two conditions, based on the pathogenic evidence |
| 18 | **Xi Xu et al. (2020 Feb 28) [17]** | Normal leucocytes 68 (76%) | Increased CRP 38 (42%), normal CRP 52 (58%) | - | - | HTN 17 (19%), DM 5 (6%), CVD 3 (3%), COPD 1 (1%) | - | - | - | All patients with laboratory-identified SARS-CoV-2 infection by real-time polymerase chain reaction (PCR) were collected between January 23, 2020, and February 4, 2020, in a designated hospital (Guangzhou Eighth People's Hospital). |
| 19 | **Chun Shuang Guan et al. (2020 Mar 6) [18]** | - | - | - | - | - | - | - | - | A total of 53 patients diagnosed with COVID-19 from January 12 to February 28, 2020, were enrolled. |
| 20 | **K. Wang et al. (2020 Mar 4) [19]** | - | - | - | - | HTN 33/114 (28.9%), CVD 7 (6.1%) | - | - | - | The study comprised 114 patients with confirmed COVID-19 treated at Xiaogan Hospital from 25 January 2020, to 9 February 2020, with complete medical records. |
| 21 | **Wanbo Zhu et al. (2020 Mar 10) [20]** | - | CRP 20.7±24.0 (0.5-112), ESR 42.4±33.6 (6-119) | - | - | HTN 22/116 (19%), DM 10 (9%), COPD 6 (5%), CVD 5 (4%) | - | - | - | This retrospective study focused on the initial clinical features of patients with suspected COVID-19 who presented to the ED of the First Affiliated Hospital of USTC and the Infectious Hospital of the First Affiliated Hospital of USTC for the first time between 24 January 2020 and 20 February 2020. |
| 22 | **Pan F et al. (2020 Feb 13) [21]** | WBC 4.9±1.0 (3.1-6.9), Neutrophil 3.1±0.8 (2.1-4.6), Lymphocyte 1.4±0.5 (0.7-2.5) | CRP 17.2±20.0 (3.1-88.6), ESR 33±27 (5-93) | | AST 42±31 (12-107), ALT 32±20 (15-95), LDH 242±73 (156-377), D-dimer 0.93±1.43 (0.17-4.70) | N/A | Mean hospitalization: 17±4 (11-26) Mild pneumonia 21/21 (100%). | Oxygen Tx 0/21, Mechanical ventilation 0/21 (excluded) | N/A | Patients diagnosed with COVID-19 pneumonia were reviewed retrospectively for the period from 12 January 2020 to 6 February 2020 in this single center study. Patients with severe pneumonia (SpO2 <90% or RR >30 or oxygen/mechanical ventilator required) during the disease course were excluded. |
| 23 | **Huang G et al. (2020 Mar 30) [22]** | - | - | - | - | N/A | - | - | - | Thirty patients were admitted to Wuhu Second People's Hospital with confirmed COVID-19 from January 22, 2020, through February 28, 2020. Patients who did not have positive CT findings or who had not yet met the conditions for discharge from the hospital were excluded from the study. |
| 24 | **Wang D et al. (2020 Mar 17) [23]** | Median (IQR) WBC 4.5 (3.3-6.2), Neutrophil 3.0 (2.0-4.9), Lymphocyte 0.8 (0.6-1.1) | N/A | | Median (IQR) AST 31 (24-51), ALT 24 (16-40), TB 9.8 mmol/L (8.4-14.1), BUN 4.4 mmol/L (3.4-5.8), Cr 72 umol/L (60-87), CK 92 (56-130), CK-MB 14 (10-18), LDH 261 (182-403), D-dimer 203 (121-403), PT 13.0 (12.3-13.7), | Total 64 (46.4%). HTN 43 (31.2%), CVD 20 (14.5%), DM 14 (10.1%), Malignancy 10 (7.2%), Cerebrovascular disease 7 (5.1%), COPD 4 (2.9%), CKD 4 (2.9%), Chronic liver disease 4 (2.9%), HIV infection 2 (1.4%) | ICU 36/138 (26.1%), median time of symptom to ICU 10 (IQR 6-12), median admission to ICU 1 (IQR 0-3) ARDS 27/138 (19.6%), median time of onset to ARDS 8.0 (IQR 6.0-12.0) | Antiviral Tx 124/138 (89.9%), Glucocorticoid therapy 62/138 (44.9%), CRRT 2/138 (1.45%), Oxygen inhalation 106/138 (76.8%), NIV 15/138 (10.9%), IMV 17/138 (12.3%), ECMO 4/138 (2.9%) | Complications: Shock 12/138 (8.7%), Acute cardiac injury 10/138 (7.2%), Arrhythymia 23/138 (16.7%), ARDS 27/138 (19.6%), AKI 5/138 (3.6%) | - |

| | | | | | | | | | | |
|---|---|---|---|---|---|---|---|---|---|---|
| | | | | | aPTT 31.4 (29.4-33.5) | | | | | |
| 25 | **Xiaoyu Han et al. (2020 Apr 17) [24]** | leukocyte 4.6±1.9, lymphocyte 1.2±0.4 | CRP 11.7 (11.8), ESR 34 (16) *(interquartile range) | | AST 33 (24.5), 31 (22) *(interquartile range) | COPD 1/17 (5%), HTN (5%), type 2 DM 2/17 (11%) | mean treatment time : 22 (18-31) | anti-viral drug, empirical antibacterial drug, abidol hydrochloride | N/A | - |
| 26 | **Shen Q et al. (2020) [25]** | WBC elevation 1/9 (11%), Lymphocyte elevation 1/9 (11%) | CRP elevation 2/9 (22%), ESR elevation 1/9 (11%) | | LDH elevation 0/9 (0%), AST elevation 2/9 (22%) | - | | Oxygen Tx 9/9, ICU care 0/9, mechanical vent 0/9 | Severe Cx 0/9 | all hospitalized children diagnosed with COVID-19 between January 8, 2019 and February 19, 2020, in Changsha Public Health Clinic Center |
| 27 | **Wu Y et al. (2020) [26]** | - | - | - | - | - | Moderate 5, Severe 6, Critical 2 | Plasminogen Tx 13/13 | | A total of 219 patients with both positive COVID-19 by RT-PCR and abnormal chest CT findings were retrospectively identified from 7 Chinese hospitals in Hunan Providence, China from January 6 to February 20, 2020. A total of 205 patients with positive Respiratory Pathogen Panel for viral pneumonia and CT findings consistent with or highly suspicious for pneumonia by original radiology interpretation within 7 days of each other were identified from Rhode Island Hospital in Providence, RI. |
| 28 | **Gao L et al. (2020) [27]** | - | - | - | - | - | - | - | - | - |
| 29 | **Shi H et al. (2020) [28]** | - | - | - | AST 46.2±29.5, ALT 40.8±17.9 | Any 21(26%), chronic pulmonary disease 9(11%), DM 10(12%), HTN 12(15%), CKD 3(4%), cardiovascular disease 8(10%), cerebrovascular disease 6(7%), malignancy 4(5%), hepatitis or liver cirrhosis 7(9%) | - | - | - | - |
| 30 | **Zhao W et al. (2020 Feb) [29]** | - | - | - | - | Any 30 (29.7%), Cardio/cerebrovascular 16 (15.8%), surgical Hx 7 (6.9%), GI disease 6 (5.9%), respiratory disease 5 (4.9%), endocrine disease 3 (3.0%) | - | - | - | Data on 101 cases of COVID-19 pneumonia were retrospectively collected from four institutions in Hunan, China. Basic clinical characteristics and detailed imaging features were evaluated and compared between two groups on the basis of clinical status: nonemergency (mild or common disease) and emergency (severe or fatal disease). |
| 31 | **Zhou Y et al. (2020 Mar) [30]** | - | - | - | - | - | aggravated 5, non-aggravated 12 | ICU, mechanical vent 0/17 | no severe Cx | - |
| 32 | **Wang Y et al. (2020) [31]** | - | - | - | - | - | Discharged 70 (78%), In admission 17 (19%), Died 2 (2%), Transferred 1 (1%) | - | - | - |

Abbreviation : COVID-19: Coronavirus disease 2019; SARS-CoV-2: severe acute respiratory syndrome–coronavirus 2; CBC: Complete blood cell count; WBC : white blood cell; CRP : C-reactive protein; ESR : erythrocyte sedimentation rate; IL: interleukin; AST: aspartate transaminase; ALT: alanine transferase; BUN: blood urea nitrogen; Cr: creatinine; LDH: lactate dehydrogenase; CK: creatine kinase; TB: total bilirubin; Glc: glucose; PT: prothrombin; PTT: partial thromboplastin time; RT-PCR : reverse transcription-polymerase chain reaction; CT : computer tomography; DM: diabetes mellitus; COPD: chronic obstructive pulmonary disease; HTN: hypertension; CVD: cardiovascular disease; CKD: chronic kidney disease; GI: gastrointestinal; HIV: human immunodeficiency virus; ARDS : acute respiratory distress syndrome; Tx: therapy; Cx: complication; Hx: history; ICU: intensive care unit; CRRT: continuous renal replacement therapy; NIV: non-invasive ventilation; IMV: intermittent mandatory ventilation; ECMO: Extracorporeal membrane oxygenation; N/A: non-available; (-): no information

**Reference**


1. Zhao D, Yao F, Wang L, Zheng L, Gao Y, Ye J, Guo F, Zhao H, Gao R. A comparative study on the clinical features of COVID-19 pneumonia to other pneumonias. Clin Infect Dis 2020 Mar 12 pii: ciaa247 doi: 101093/cid/ciaa247 Epub ahead of print
2. Caruso D, Zerunian M, Polici M, Pucciarelli F, Polidori T, Rucci C, Guido G, Bracci B, de Dominicis C, Laghi A. Chest CT Features of COVID-19 in Rome, Italy. Radiology 2020 Apr 3:201237 doi: 101148/radiol2020201237 Epub ahead of print
3. Bernheim A, Mei X, Huang M, Yang Y, Fayad ZA, Zhang N, Diao K, Lin B, Zhu X, Li K, Li S, Shan H, Jacobi A, Chung M. Chest CT Findings in Coronavirus Disease-19 COVID-19: Relationship to Duration of Infection Radiology 2020 Feb 20:200463 doi: 101148/radiol2020200463 Epub ahead of print
4. Wu J, Wu X, Zeng W, Guo D, Fang Z, Chen L, Huang H, Li C. Chest CT Findings in Patients With Coronavirus Disease 2019 and Its Relationship With Clinical Features. Invest Radiol 2020 May;555:257-261 doi: 101097/RLI0000000000000670
5. Xu YH, Dong JH, An WM, Lv XY, Yin XP, Zhang JZ, Dong L, Ma X, Zhang HJ, Gao BL. Clinical and computed tomographic imaging features of novel coronavirus pneumonia caused by SARS-CoV-2. J Infect 2020 Apr;804:394-400 doi: 101016/jjinf202002017 Epub 2020 Feb 25

6. Ai T, Yang Z, Hou H, Zhan C, Chen C, Lv W, Tao Q, Sun Z, Xia L. Correlation of Chest CT and RT-PCR Testing in Coronavirus Disease 2019 COVID-19 in China: A Report of 1014 Cases. Radiology 2020 Feb 26:200642 doi: 101148/radiol2020200642 Epub ahead of print
7. Zhou S, Wang Y, Zhu T, Xia L. CT Features of Coronavirus Disease 2019 COVID-19 Pneumonia in 62 Patients in Wuhan, China AJR Am J Roentgenol 2020 Mar 5:1-8 doi: 102214/AJR2022975 Epub ahead of print
8. Zhang R, Ouyang H, Fu L, Wang S, Han J, Huang K, Jia M, Song Q, Fu Z. CT features of SARS-CoV-2 pneumonia according to clinical presentation: a retrospective analysis of 120 consecutive patients from Wuhan city. Eur Radiol 2020 Apr 11 doi: 101007/s00330-020-06854-1 Epub ahead of print
9. Li K, Fang Y, Li W, Pan C, Qin P, Zhong Y, Liu X, Huang M, Liao Y, Li S. CT image visual quantitative evaluation and clinical classification of coronavirus disease COVID-19. Eur Radiol 2020 Mar 25 doi: 101007/s00330-020-06817-6 Epub ahead of print
10. Chung M, Bernheim A, Mei X, Zhang N, Huang M, Zeng X, Cui J, Xu W, Yang Y, Fayad ZA, Jacobi A, Li K, Li S, Shan H. CT Imaging Features of 2019 Novel Coronavirus 2019-nCoV. Radiology 2020 Apr;2951:202-207 doi: 101148/radiol2020200230 Epub 2020 Feb 4
11. Liu KC, Xu P, Lv WF, Qiu XH, Yao JL, Gu JF, Wei W. CT manifestations of coronavirus disease-2019: A retrospective analysis of 73 cases by disease severity. Eur J Radiol 2020 Mar 12;126:108941 doi: 101016/jejrad2020108941 Epub ahead of print
12. Long C, Xu H, Shen Q, Zhang X, Fan B, Wang C, Zeng B, Li Z, Li X, Li H. Diagnosis of the Coronavirus disease COVID-19: rRT-PCR or CT? Eur J Radiol 2020 Mar 25;126:108961 doi: 101016/jejrad2020108961 Epub ahead of print
13. Himoto Y, Sakata A, Kirita M, Hiroi T, Kobayashi KI, Kubo K, Kim H, Nishimoto A, Maeda C, Kawamura A, Komiya N, Umeoka S. Diagnostic performance of chest CT to differentiate COVID-19 pneumonia in non-high-epidemic area in Japan. Jpn J Radiol 2020 Mar 30 doi: 101007/s11604-020-00958-w Epub ahead of print
14. Han R, Huang L, Jiang H, Dong J, Peng H, Zhang D. Early Clinical and CT Manifestations of Coronavirus Disease 2019 COVID-19 Pneumonia. AJR Am J Roentgenol 2020 Mar 17:1-6 doi: 102214/AJR2022961 Epub ahead of print
15. Zhang X, Cai H, Hu J, Lian J, Gu J, Zhang S, Ye C, Lu Y, Jin C, Yu G, Jia H, Zhang Y, Sheng J, Li L, Yang Y. Epidemiological, clinical characteristics of cases of SARS-CoV-2 infection with abnormal imaging findings. Int J Infect Dis 2020 Mar 20 pii: S1201-97122030172-7 doi: 101016/jijid202003040 Epub ahead of print
16. Dai H, Zhang X, Xia J, Zhang T, Shang Y, Huang R, Liu R, Wang D, Li M, Wu J, Xu Q, Li Y. High-resolution Chest CT Features and Clinical Characteristics of Patients Infected with COVID-19 in Jiangsu, China. Int J Infect Dis 2020 Apr 6 pii: S1201-97122030218-6 doi: 101016/jijid202004003 Epub ahead of print
17. Xu X, Yu C, Qu J, Zhang L, Jiang S, Huang D, Chen B, Zhang Z, Guan W, Ling Z, Jiang R, Hu T, Ding Y, Lin L, Gan Q, Luo L, Tang X, Liu J. Imaging and clinical features of patients with 2019 novel coronavirus SARS-CoV-2 Eur J Nucl Med Mol Imaging 2020 May;475:1275-1280 doi: 101007/s00259-020-04735-9 Epub 2020 Feb 28
18. Guan CS, Lv ZB, Yan S, Du YN, Chen H, Wei LG, Xie RM, Chen BD. Imaging Features of Coronavirus disease 2019 COVID-19: Evaluation on Thin-Section CT. Acad Radiol 2020 Mar 20 pii: S1076-63322030143-4 doi: 101016/jacra202003002 Epub ahead of print
19. Wang K, Kang S, Tian R, Zhang X, Zhang X, Wang Y. Imaging manifestations and diagnostic value of chest CT of coronavirus disease 2019 COVID-19 in the Xiaogan area. Clin Radiol 2020 May;755:341-347 doi: 101016/jcrad202003004 Epub 2020 Mar 23
20. Zhu W, Xie K, Lu H, Xu L, Zhou S, Fang S. Initial clinical features of suspected coronavirus disease 2019 in two emergency departments outside of Hubei, China. J Med Virol 2020 Mar 13 doi: 101002/jmv25763 Epub ahead of print
21. Pan F, Ye T, Sun P, Gui S, Liang B, Li L, Zheng D, Wang J, Hesketh RL, Yang L, Zheng C. Time Course of Lung Changes On Chest CT During Recovery From 2019 Novel Coronavirus COVID-19 Pneumonia. Radiology 2020 Feb 13:200370 doi: 101148/radiol2020200370 Epub ahead of print
22. Huang G, Gong T, Wang G, Wang J, Guo X, Cai E, Li S, Li X, Yu Y, Lin L. Timely Diagnosis and Treatment Shortens the Time to Resolution of Coronavirus Disease COVID-19 Pneumonia and Lowers the Highest and Last CT Scores From Sequential Chest CT. AJR Am J Roentgenol 2020 Mar 30:1-7 doi: 102214/AJR2023078 Epub ahead of print
23. Wang D, Hu B, Hu C, Zhu F, Liu X, Zhang J, Wang B, Xiang H, Cheng Z, Xiong Y, Zhao Y, Li Y, Wang X, Peng Z. Clinical Characteristics of 138 Hospitalized Patients with 2019 Novel Coronavirus-Infected Pneumonia in Wuhan, China. JAMA - Journal of the American Medical Association 2020 323:11 1061-1069 Date of Publication: 17 Mar 2020
24. Han X, Cao Y, Jiang N, Chen Y, Alwalid O, Zhang X, Gu J, Dai M, Liu J, Zhu W, Zheng C, Shi H. Novel Coronavirus Pneumonia COVID-19 Progression Course in 17 Discharged Patients: Comparison of Clinical and Thin-Section CT Features During Recovery. Clin Infect Dis 2020 Mar 30 pii: ciaa271 doi: 101093/cid/ciaa271 Epub ahead of print
25. Shen Q, Guo W, Guo T, Li J, He W, Ni S, Ouyang X, Liu J, Xie Y, Tan X, Zhou Z, Peng H. Novel coronavirus infection in children outside of Wuhan, China Pediatr Pulmonol 2020 Apr 7 doi: 101002/ppul24762 Epub ahead of print
26. Wu Y, Wang T, Guo C, Zhang D, Ge X, Huang Z, Zhou X, Li Y, Peng Q, Li J. Plasminogen improves lung lesions and hypoxemia in patients with COVID-19. QJM 2020 Apr 10 pii: hcaa121 doi: 101093/qjmed/hcaa121 Epub ahead of print
27. Gao L, Zhang J. Pulmonary High-Resolution Computed Tomography HRCT Findings of Patients with Early-Stage Coronavirus Disease 2019 COVID-19 in Hangzhou, China. Med Sci Monit 2020 Apr 4;26:e923885 doi: 1012659/MSM923885
28. Shi H, Han X, Jiang N, Cao Y, Alwalid O, Gu J, Fan Y, Zheng C. Radiological findings from 81 patients with COVID-19 pneumonia in Wuhan, China: a descriptive study. Lancet Infect Dis 2020 Apr;204:425-434 doi: 101016/S1473-30992030086-4 Epub 2020 Feb 24
29. Zhao W, Zhong Z, Xie X, Yu Q, Liu J. Relation Between Chest CT Findings and Clinical Conditions of Coronavirus Disease COVID-19 Pneumonia: A Multicenter Study. AJR Am J Roentgenol 2020 Mar 3:1-6 doi: 102214/AJR2022976 Epub ahead of print
30. Zhou Y, Zhang Z, Tian J, Xiong S. Risk factors associated with disease progression in a cohort of patients infected with the 2019 novel coronavirus. Ann Palliat Med 2020 Mar;92:428-436 doi: 1021037/apm20200326 Epub 2020 Mar 17
31. Wang Y, Dong C, Hu Y, Li C, Ren Q, Zhang X, Shi H, Zhou M. Temporal Changes of CT Findings in 90 Patients with COVID-19 Pneumonia: A Longitudinal Study. Radiology 2020 Mar 19:200843 doi: 101148/radiol2020200843 Epub ahead of print
